# Emerging trends and clinical recommendations for zirconia ceramic crowns: a concise review

**DOI:** 10.1038/s41415-024-7616-0

**Published:** 2024-07-12

**Authors:** Karishma Vijan

**Affiliations:** Alumini University of Manchester, Manchester, UK

## Abstract

**Introduction** A little over ten years ago, zirconia crowns made their debut in the field of dentistry. Despite early problems with the chipping of veneering porcelain, clinical studies have demonstrated excellent performance. It is essential for a ceramic crown to have good aesthetic qualities, in addition to having good mechanical characteristics. The exceptional mechanical qualities of zirconia crowns and the simplicity with which they may be machined, employing computer-aided design and computer-aided manufacturing schemes, are primarily responsible for the widespread use of these materials in clinical settings. New ceramic-based materials, including monolithic zirconia, zirconia-containing lithium disilicate ceramics, and graded glass/zirconia/glass, have recently been launched in the field of dentistry. These newly discovered zirconia crown materials stem from varied technological approaches, each likely to lead to additional clinical advancements. At this point, it seems imperative to offer a concentrated report on the newer developments, along with essential clinical recommendations for best clinical outcomes with zirconia crowns.

**Types of studies** This review article is a consolidation of several case studies, cohort studies and systematic reviews, as well as experimental and observational randomised control trials and other peer-reviewed articles.

**Results** On reviewing, a concise list of clinical recommendations is generated, demonstrating that monolithic zirconia offers some clinical advantages over veneered zirconia crowns.

**Conclusion** This review article discloses various clinical revelations and in-office recommendations for favourable usage of zirconia ceramic crowns that can lead to better patient outcomes and long-term clinical success rates.

## Key points

- To master clinical knowledge with novel insights on zirconia crowns.
- To grasp current trends and methodology for long-term clinical success with zirconia ceramics.
- To gain scientific expertise through clinical guidelines for long-standing restorative success.
- To learn new innovations and trends with zirconia crowns and restorations.

## Background

Zirconia crowns first appeared in dentistry a little more than ten years ago. Despite early problems with the chipping of veneering porcelain, clinical studies have demonstrated excellent performance.^1^ It is essential for a ceramic crown to have good aesthetic qualities, in addition to having good mechanical characteristics.^2^^,^^3^ The exceptional mechanical properties of zirconia crowns, as well as the ease with which they can be machined using computer-aided design and computer-aided manufacturing (CAD/CAM) schemes, are largely responsible for their widespread use in clinical settings. New ceramic-based materials, including monolithic zirconia, zirconia-containing lithium disilicate ceramics (ZLCs), and graded glass/zirconia/glass (GZG), have recently been launched in the field of dentistry. These newly discovered zirconia crown materials stem from varied technological approaches, each likely to lead to additional clinical advancements. At this point, it appears necessary to provide a focused report on the most recent developments, as well as critical clinical recommendations for the best clinical outcomes with zirconia crowns.^4^

## Introduction

Zirconia, also known as zirconium dioxide (ZrO_2_), is found in its most natural form in the mineral baddeleyite. For more than a decade, zirconia ceramics have become an integral part of dentistry.^4^ Despite preliminary issues, such as chipping of veneering porcelain, scientific studies have elaborated on their optimum performance.^4^ A classic ceramic restorative material must have distinguishing mechanical and aesthetic properties.^5^ Zirconia's clinical recognition and triumph can be attributed to its remarkable mechanical properties and CAD/CAM ease. In recent times, novel ceramic materials have emerged in dentistry, such as the monolithic zirconia, ZLCs and graded zirconia. These upcoming restorative materials are derived from unique technologies that have the potential to lead to further advancements. It is therefore critical to place a brief emphasis on these new materials, as well as their mechanical, aesthetic and clinical properties.

## Evolution of zirconia ceramics

The prosthetic dentistry field is experiencing a shift in its approach, moving away from the use of metal-ceramic restorations and towards the utilisation of all-ceramic prostheses. This shift is primarily driven by the desire to improve both the aesthetic appearance and biocompatibility of prosthetic dental devices. Ceramic materials exhibit greater resistance to biological degradation compared to their metallic counterparts, making them a crucial consideration for implant applications.^5^ The issue of tooth wear merits attention, considering the unusual hardness of zirconia. The reduction of abrasive wear on opposing tooth enamel can be achieved by producing highly polished surfaces. Though possessing desirable properties, ceramic materials exhibit a tendency towards fragility and susceptibility to fracture. Hence, there has been a recent emphasis on the advancement of ceramic materials that possess both robustness and visual appeal. In dentistry, it has been customary to employ zirconia composed predominantly of small tetragonal crystals of zirconia, stabilised with a restricted quantity (3 mol%) of yttrium, called yttrium-stabilised tetragonal zirconia polycrystals (3Y-TZP). Despite their inherent weakness, the attainment of the ideal levels of translucency and opalescence in lithia-based glass ceramics remains an unresolved matter. Lithia-based glass-ceramic zirconias continue to be the favoured material for anterior prostheses.^6^ However, it is important to note that the enhancement of both aesthetic characteristics and mechanical durability is an ongoing effort.^7^ Recently, there has been a growing demand for novel Y-TZP zirconias with graded and nanoscale microstructures being considered as a crucial option for anterior prosthes.^6^

Examining the advancement of ceramic materials within the framework of clinical practice is worthwhile. Although possessing exceptional strength, ceramics made of 3Y-TZP exhibit suboptimal translucency. The use of materials with high translucency, by increasing the yttria content to 4 mol% or 5 mol% (4Y-PSZ and 5Y-PSZ), are thereby partially stabilising zirconia (PSZ). The use of materials with high translucency, which include 5Y-PSZ, is restricted to single-unit crowns, as well as short-span fixed dental prostheses for the anterior region.^8^ Optimising the mechanical properties of materials is crucial in fully leveraging the ultra-translucent nature of 5Y-PSZ.^8^

## Monolithic zirconia

Several researchers suggest using monolithic Y-TZP crowns to mitigate veneer fractures. According to scientific research, monolithic Y-TZP has been recommended as a suitable solution for addressing structural demands associated with posterior applications involving higher forces. Notably, Y-TZP demonstrates considerably elevated levels of stability and elastic modulus of synthetic materials is often compared to that of natural dentine. The variability in tooth values may lead to disproportionate attrition of the opposing dentition; the harder materials may cause wear of the enamel of the opposing tooth.^9^^,^^10^

## Pressed zirconia

Contemporary materials such as GZG have the ability to provide both wear resistance and aesthetic appeal. Using analytical techniques, it is possible to develop GZG materials that exhibit superior strength and resistance to contact damage compared to zirconia. Furthermore, these materials can maintain surface physical and optical characteristics analogous to porcelain. Further clarification is necessary to account for the notably elevated resistance to damage caused by sliding contact in the graded zirconia-glass composite material. It is the ‘over-pressing technique' that has been contemplated. The structural framework of zirconia is coated with a specialised ceramic through a pressing process. As per Beuer *et al.*'s findings, this technique's predictability can be gauged to the absence of any chipping occurrences.^11^ The utilisation of the pressing technique enables the creation of intended dental morphology while mitigating the effects of firing shrinkage.^12^ In a novel study, layered ceramics had significantly more fractures than over-pressed zirconia three-unit posterior prostheses.^13^ However, in another study, no instances of chipping were observed.^14^^,^^15^ Ishibe and Aboushelib proposed the utilisation of press-on veneer ceramics on airborne-particle-abraded surfaces.^16^^,^^17^^,^^18^^,^^19^ Nevertheless, several studies have reported a lack of consensus regarding the incidence of fractures between the pressed and layered.^14^^,^^15^^,^^20^

## Zirconia-containing lithium silicate ceramics

The advancement of lithium silicate glass ceramics containing zirconia represents the progressive pursuit of ceramic materials that include both effective translucency and outstanding mechanical characteristics. The types of crystalline phases present in ZLCs, including lithium metasilicate and lithium disilicate for ZLCs, are the primary cause of the fundamental difference that can be seen in the final crystallisation phase of lithium disilicate glass ceramics in comparison to ZLCs. The durability of these ceramics may potentially surpass that of zirconia ceramics. Dental ceramics have been refined for aesthetic purposes, leading to the development of ceramic-glass and ceramic-polymer interpenetrating phase composites for use in dental medicine - a different group of material requiring discussion separately.

## New class of submicron grain-sized alumina ceramics

**Fig. 1:**
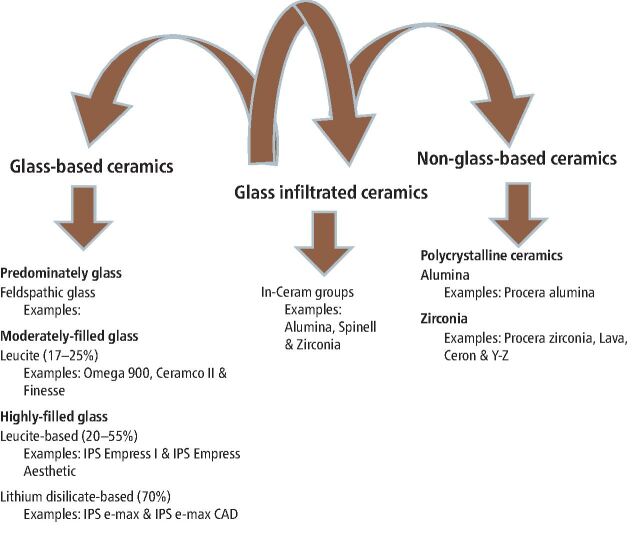
Classifications of ceramics according to their composition with examples of commercially available ceramic types. Reprinted from *The Saudi Dental Journal*, vol 32, Warreth *et al.*, ‘All-ceramic restorations: A review of the literature', pp 365-372, copyright 2020, with permission from Elsevier^52^

When compared to both cubic-containing zirconia and lithium disilicate glass-ceramic, the recently manufactured submicron polycrystalline aluminas display a higher level of translucency. The degree of translucency exhibited is comparable to that of high translucency ceramics and ceramic coarse-grained aluminas (CGAs) that are available in the market, and it is analogous to commercially available CGA. When it comes to the photopolymerisation of luting cement, the greater Transmitted Irradiance Time (TIT) value that the submicron aluminas have in the short wavelength region is helpful. In comparison to zirconias with cubic structures and glass ceramics based on lithia, the submicron aluminas have a higher strength profile. Alumina's slow crack-growth velocity exponent is comparable to that of zirconia; however, unlike zirconia, alumina is not vulnerable to low-temperature deterioration. Consequently, the recently created submicron aluminas may demonstrate resistance that is superior to that of glass ceramics and zirconia. Because of this, they are an excellent material for fabricating dental crowns. The newly developed submicron polycrystalline aluminas have a degree of translucency comparable to that of high-translucency porcelains. Furthermore, the aforementioned alumina ceramics have exhibited considerably higher strength in comparison to lithium disilicate glass-ceramic and zirconia having a cubic phase (Fig. 1).

## Graded zirconia

**Fig. 2:**
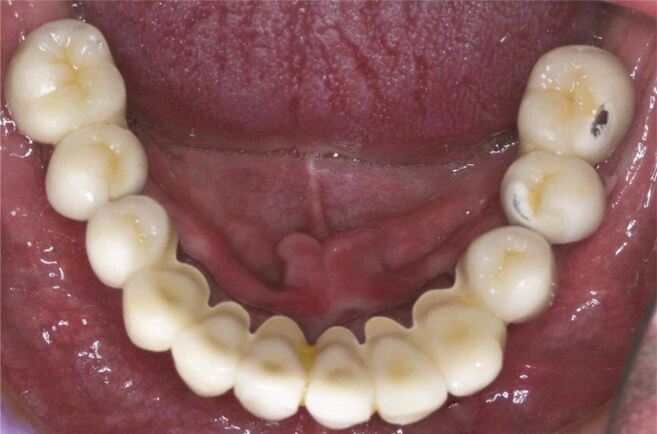
Chipping fractures in porcelain-veneer dental prosthesis. Reprinted from *The Journal of Prosthetic Dentistry*, vol 122, Papaspyridakos *et al*., ‘Complications and survival rates of 55 metal-ceramic implant-supported fixed complete-arch prostheses: A cohort study with mean 5-year follow-up', pp 441-449, copyright 2019, with permission from Elsevier^53^

The graded zirconia glass material offers a solution that is both aesthetically acceptable and damage-resistant for posterior dental crowns. They are also a good fit for restorations such as onlays, inlays, crowns, Maryland bridges and fixed partial dentures (FPDs). A straightforward staining method or applying a ceramic overlay in a very thin layer may accomplish this objective. The chipping and fracture of veneers are commonly found as failure causes in porcelain-veneered zirconia dental crowns (Fig. 2). The usage of GZG with external aesthetic glass (e-GZG), as indicated by certain estimates, may increase the durability and give higher protection against veneer chipping and fracture in comparison to porcelain veneered zirconia while still keeping the essential aesthetic attributes.^21^ A graded GZG structurep with porcelain-veneered zirconia systems displayed a much lower level of reaffirmed resistance against sliding contact fatigue than the graded GZG structure with an exterior glass material that was visually acceptable.^21^ This innovative GZG with external aesthetic glass e-GZG structure has the ability to make it easier to meet the aesthetic requirements for dental restorations. The opaque and artificially white look of the monolithic Y-TZP is successfully contrasted by the fact that the exterior glass layer displays outstanding translucency and provides a variety of shade choices. In addition, the colour of the e-GZG may be altered by adjustments in the glass's composition, making it a versatile material. Incorporating a translucent Y-TZP into the composition of the material is another way to increase the level of transparency possessed by the material. The e-GZG has a glassy surface and a lower modulus and hardness than other materials. The property, as mentioned above, serves as a protective barrier against excessive abrasion that the rigid and homogeneous Y-TZP material on the opposing dentition may cause. Furthermore, an evaluation has been conducted on the material‘s capacity to withstand contact and flexural damage, and a technique for glass-ceramic infiltration was developed to maintain the materials structural soundness.^12^ The graded glass-zirconia structure located at the cementation surface of zirconia mitigates bending stresses, consequently enhancing zirconia's flexural structure. The graded structure can offer resistance against fatigue sliding damage due to its inherent design.

## Newer zirconia materials (ultra translucent)

Materials made of zirconia that have a high level of translucency are gaining a lot of attention these days owing to their exceptional aesthetic attributes. The increased cubic zirconia concentration results in a decrease in the material's strength, as well as its capacity to undergo transformation toughening. This is the case even if the material has high translucency. When contrasted with the highly polished 5Y-PSZ, which has a strength of (467 38 MPa), the ultra-translucent glass-infiltrated 5Y-PSZ demonstrates an increase in strength by 25%. Additionally, it has been noted that 5Y-PSZ's translucency, which has been measured to have a translucency parameter (TP) of 34 as well as a contrast ratio (CR) of 0.31, does not change even after glass infiltration (TP = 34; CR = 0.32), provided that the remaining surface of glass is removed using a delicate polishing procedure that uses 6 m and 3 m diamond grits.^22^^,^^23^ The glass-infiltrated 5Y-PSZ, which has only recently achieved its maturity stage, boasts a unique combination of high strength and translucency, making it an exciting candidate for next-generation crowns for teeth that are both damage-resistant and aesthetically attractive.

## Failures of zirconia

**Fig. 3:**
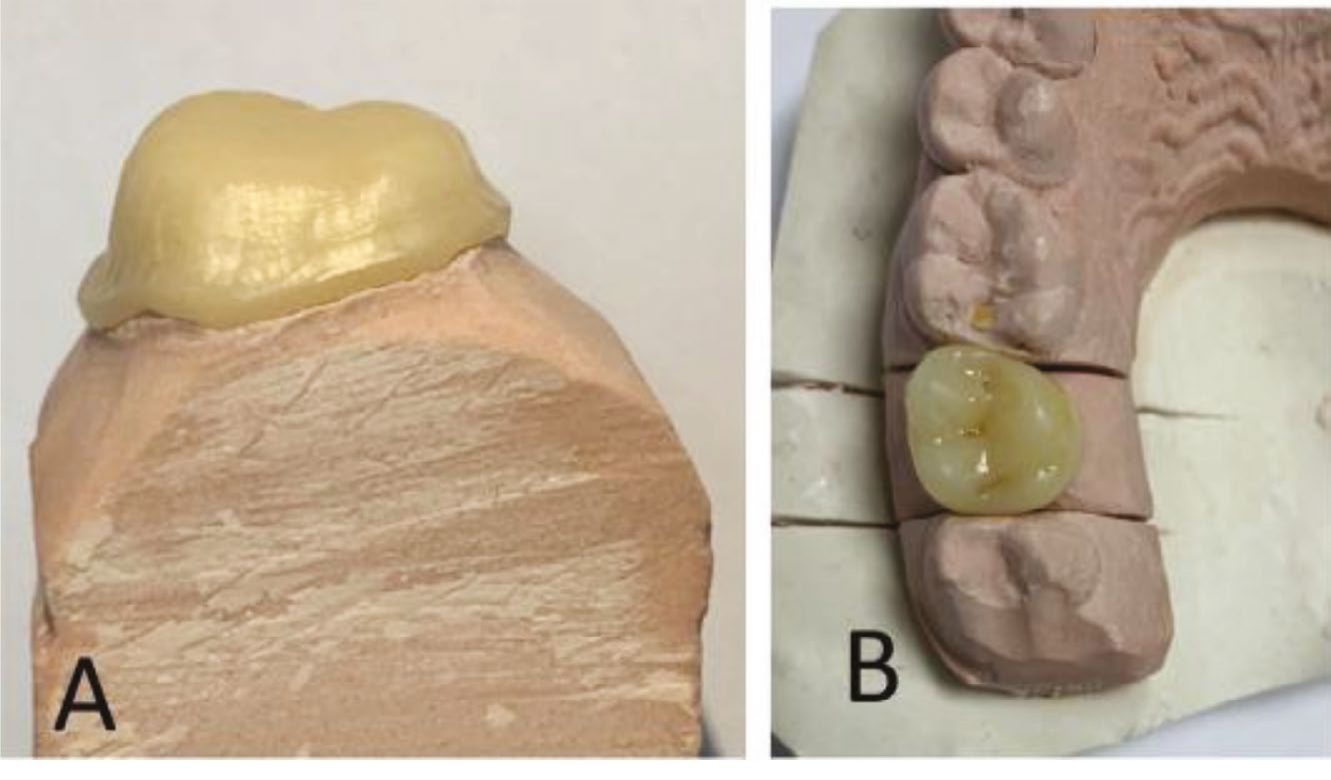
a) An all-ceramic core on a die stone. b) The same core veneered with a high glass-ceramic. Reprinted from *The Saudi Dental Journal*, vol 32, Warreth *et al*., ‘All-ceramic restorations: A review of the literature', pp 365-372, copyright 2020, with permission from Elsevier^52^

The shortcomings of all-ceramic crowns based on zirconia frequently manifest as notable fractures and chips in the porcelain veneer due to sliding contact damage resulting from occlusal stresses. Scientific studies that included the incorporation of glass into zirconia plates produced graded structures, which led to improved aesthetic characteristics and higher modulus at the surfaces (Figure 3). A study showed the graded structures exhibited a resistance that was over 25 times stronger compared to that of veneered zirconia and over three times higher than those of monolithic zirconia.^24^ When compared to homogeneous zirconia, the cementation properties of the zirconia-glass materials are superior, and the zirconia-glass materials display a range of hues from white to yellow.

## Clinical recommendations

We are aware that in contrast to metal-ceramic crowns, zirconia crowns boast a simplified manufacturing process thanks to digital technology.^25^^,^^26^ CAD/CAM software replaces intricate wax carving and casting, while the lack of a metal framework streamlines steps and minimises error.^27^ This digital approach also expedites production, translating to quicker turnaround times for patients.^28^ According to the findings of the research that was done, monolithic zirconia provides a number of benefits. Clinical advantages are associated with veneered zirconia crowns.^22^ The assessment of natural dentition wear is of major relevance throughout the process of choosing a restorative material. This is especially true within the context of behaviour that is considered parafunctional.^29^ The discussion above indicates that novel zirconia based ceramic materials have the essential qualities to meet the aesthetic and practical requirements that are being raised.

The following is a list of some of the clinical discoveries and in-office suggestions that have been made about this beneficial material:

1. Compared to glazed zirconia and glazed-veneering porcelain, polished zirconia causes enamel to wear slowly^30^
2. Occlusal adjustment techniques performed after cementation have the potential to enhance the surface roughness, which in turn might hasten the wear of tooth enamel. After occlusal alterations have been made, zirconia should have a polish applied to it to avoid this problem^31^
3. There are scientific studies suggesting that zirconia thickness can be reduced up to 0.5 mm keeping a sufficient strength to endure maximum chewing forces up to 900 N^6^^,^^12^^,^^21^^,^^32^^,^^33^^,^^34^
4. Due to the great mechanical strength of polished monolithic zirconia, it is the optimum choice for posterior FPDs in situations where clenching and grinding are present, primarily in posterior regions due to its inherent aesthetic limitations^35^^,^^36^
5. In situations with restricted occlusal crown space, monolithic zirconia crowns are advised for its superior fracture resistance when compared to veneered equivalents and other monolithic crowns^37^^,^^38^^,^^39^
6. Even at minimal thicknesses, monolithic zirconia can be successfully fabricated using a minimal quantity of material and has shown to have adequate fracture resistance at a minimum of 0.5 mm thickness as well^12^^,^^33^
7. Even if a monolithic zirconia restoration is designed for anterior use and has an advanced degree of translucency^34^
8. After evaluating different all-ceramic systems, Raigrodski concluded that reinforced zirconias are suitable only for replacing single crown restorations in anterior teeth or a maximum of three-unit FPDs for posterior regions^36^
9. In a study by Tinschert, the lifetime of various metal-free cores for FPDs was compared, and it was reported that zirconia-ceramic with alumina oxide exhibited the highest initial and most favourable long-term strength^40^^,^^41^
10. The minimum required connecting surface area for an FPD is 6.25 mm² for a three-unit posterior bridge^42^^,^^43^
11. Ceramic FPDs should be used when the distance between the interproximal papilla and the marginal ridge is approximately 4 mm^44^^,^^45^^,^^46^
12. Resin-bonded luting has been proven to be the preferred choice for zirconia-ceramic crowns and restorations, although conventional cementation may also be permissible^47^^,^^48^
13. To prevent mechanical fracture of full zirconia crowns, the thickness of the zirconia crown and proper sintering processes should be taken into consideration.^49^ The preparation of teeth should involve a reduction of at least 1.5 mm incisal/occlusal and 1.0 mm axial on the margin with a 4-6-degree taper. In cases where aesthetics is of utmost importance, the axial reduction may be increased to 1.5 mm^45^
14. Kern and Wegner highlighted that the utilisation of airborne or silane did not enhance resin bond adhesion on zirconia^44^^,^^50^
15. Tribochemical treatment appears to improve bonding. Surface treatment does not seem to be necessary to achieve satisfactory adhesion^51^

According to current research, zirconia ceramic crowns should be cemented with resin cement without surface treatment or with tribochemical treatment. The tribochemical treatment application is favoured as the surface treatment option for the ceramic restorations before luting with resin cement.^14^^,^^51^

## Conclusion

Zirconia's future, along with that of any other restorative ceramic, will be determined by basic advancements in the materials science community, followed by the development of innovative dental manufacturing techniques by enterprising dental manufacturers, and finally, the application of these techniques by trained clinical craftspeople.^12^ Improving aesthetics while preserving the superior inherent strength of material is a one-of-a-kind combination exhibited by monolithic zirconias and can be advantageous for achieving optimum clinical results.^6^^,^^32^^,^^36^

## Data Availability

The data for use in the study are available from the websites referenced in the article.

## References

[1] Denry I, Holloway J A. Ceramics for dental applications: A review. *Materials (Basel)* 2010; **3:** 351-368.

[2] Zhao M, Sun Y, Zhang J, Zhang Y. Novel Translucent and Strong Submicron Alumina Ceramics for Dental Restorations. *J Dent Res* 2018; **97:** 289-295. 10.1177/0022034517733742 PMC5833183 28977778

[3] Zhang Y, Kim J W. Graded zirconia glass for resistance to veneer fracture. *J Dent Res* 2010; **89:** 1057-1062. 10.1177/0022034510375289 PMC3318052 20651092

[4] Denry I, Kelly J R. Emerging ceramic-based materials for dentistry. *J Dent Res* 2014; **93:** 1235-1242. 10.1177/0022034514553627 PMC4237640 25274751

[5] Sailer I, Philipp A, Zembic A, Pjetursson B E, Hämmerle C H F, Zwahlen M. A systematic review of the performance of ceramic and metal implant abutments supporting fixed implant reconstructions. *Clin Oral Implants Res* 2009; **24:** 4-31. 10.1111/j.1600-0501.2009.01787.x 19663946

[6] Zhang Y, Lawn B R. Novel Zirconia Materials in Dentistry. *J Dent Res* 2018; **97:** 140-147. 10.1177/0022034517737483 PMC5784474 29035694

[7] Hannink R H J, Kelly P M, Muddle B C. Transformation Toughening in Zirconia-Containing Ceramics. *J Am Ceram Soc* 2000; **83:** 461-487.

[8] Sailer I, Pjetursson B E, Zwahlen M, Hämmerle C H F. A systematic review of the survival, complication rates of all-ceramic, metal-ceramic reconstructions after an observation period of at least 3 years. Part II: Fixed dental prostheses. *Clin Oral Implants Res* 2007; **18:** 86-96. 10.1111/j.1600-0501.2007.01468.x 17594373

[9] Tang Z, Zhao X, Wang H, Liu B. Clinical evaluation of monolithic zirconia crowns for posterior teeth restorations. *Medicine (Baltimore)* 2019; **98:** e17385. 10.1097/MD.0000000000017385 PMC6783234 31577743

[10] Denry I, Kelly J R. State of the art of zirconia for dental applications. *Dent Mater* 2008; **24:** 299-307. 10.1016/j.dental.2007.05.007 17659331

[11] Beuer F, Edelhoff D, Gernet W, Sorensen J A. Three-year clinical prospective evaluation of zirconia-based posterior fixed dental prostheses (FDPs). *Clin Oral Investig* 2009; **13:** 445-451. 10.1007/s00784-009-0249-5 19169719

[12] Daou E E. The zirconia ceramic: strengths and weaknesses. *Open Dent J* 2014; **8:** 33-42. 10.2174/1874210601408010033 PMC4026739 24851138

[13] Christensen R P, Eriksson K A, Ploeger B J. Clinical performance of PFM, zirconia, and alumina three-unit posterior prostheses. Available at http://43.230.198.52/168/ABSTRACT/A105962.htm (accessed June 2024).

[14] Stawarczyk B, Ozcan M, Roos M, Trottmann A, Sailer I, Hämmerle C H F. Load-bearing capacity and failure types of anterior zirconia crowns veneered with overpressing and layering techniques. *Dent Mater* 2011; **27:** 1045-1053. 10.1016/j.dental.2011.07.006 21820726

[15] Beuer F, Schweiger J, Eichberger M, Kappert H F, Gernet W, Edelhoff D. High-strength CAD/CAM-fabricated veneering material sintered to zirconia copings - a new fabrication mode for all-ceramic restorations. *Dent Mater* 2009; **25:** 121-128. 10.1016/j.dental.2008.04.019 18620748

[16] Aboushelib M N, Kleverlaan C J, Feilzer A J. Effect of zirconia type on its bond strength with different veneer ceramics. *J Prosthodont* 2008; **17:** 401-408. 10.1111/j.1532-849X.2008.00306.x 18355163

[17] Aboushelib M N, Kleverlaan C J, Feilzer A J. Microtensile bond strength of different components of core veneered all-ceramic restorations. Part II: Zirconia veneering ceramics. *Dent Mater* 2006; **22:** 857-863. 10.1016/j.dental.2005.11.014 16376981

[18] Ishibe M, Raigrodski A J, Flinn B D, Chung K-H, Spiekerman C, Winter R R. Shear bond strengths of pressed and layered veneering ceramics to high-noble alloy and zirconia cores. *J Prosthet Dent* 2011; **106:** 29-37. 10.1016/S0022-3913(11)60090-5 21723991

[19] Scherrer S S, Cesar P F, Swain M V. Direct comparison of the bond strength results of the different test methods: a critical literature review. *Dent Mater* 2010; **26:** 78-93. 10.1016/j.dental.2009.12.002 20060160

[20] Guess P C, Zhang Y, Thompson V P. Effect of veneering techniques on damage and reliability of Y-TZP trilayers. *Eur J Esthet Dent* 2009; **4:** 262-276. 19704927

[21] Ren L, Janal M N, Zhang Y. Sliding contact fatigue of graded zirconia with external esthetic glass. *J Dent Res* 2011; **90:** 1116-1121. 10.1177/0022034511412075 PMC3169883 21666105

[22] Malkondu Ö, Tinastepe N, Akan E, Kazazoğlu E. An overview of monolithic zirconia in dentistry. *Biotechnol Biotechnol Equip* 2016; **30:** 644-652.

[23] Mao L, Kaizer M R, Zhao M, Guo B, Song Y F, Zhang Y. Graded Ultra-Translucent Zirconia (5Y-PSZ) for Strength and Functionalities. *J Dent Res* 2018; **97:** 1222-1228. 10.1177/0022034518771287 PMC6151910 29694258

[24] Güncü M B, Cakan U, Muhtarogullari M, Canay S. Zirconia-based crowns up to 5 years in function: a retrospective clinical study and evaluation of prosthetic restorations and failures. *Int J Prosthodont* 2015; **28:** 152-157. 10.11607/ijp.4168 25822300

[25] Raigrodski A J. Contemporary materials and technologies for all-ceramic fixed partial dentures: a review of the literature. *J Prosthet Dent* 2004; **92:** 557-562. 10.1016/j.prosdent.2004.09.015 15583562

[26] Gulati M, Anand V, Salaria S K, Jain N, Gupta S. Computerized implant-dentistry: Advances toward automation. *J Indian Soc Periodontol* 2015; **19:** 5-10. 10.4103/0972-124X.145781 PMC4365158 25810585

[27] Di Rocco D. Use of the CEREC system in dental practices. Esthetic reconstruction of the anterior teeth--a case report. *Schweiz Monatsschr Zahnmed* 2009; **119:** 717-729. 19694191

[28] Wu L, Sun Z, Zhao J, Zheng Y. Retrospective clinical study of monolithic zirconia crowns fabricated with a straightforward completely digital workflow. *J Prosthet Dent* 2022; **128:** 913-918. 10.1016/j.prosdent.2021.01.018 33678440

[29] Paryag A, Rafeek R. Dental Erosion and Medical Conditions: An Overview of Aetiology, Diagnosis and Management. *West Indian Med J* 2014; **63:** 499-502. 10.7727/wimj.2013.140 PMC4655683 25781289

[30] Janyavula S, Lawson N, Cakir D, Beck P, Ramp L C, Burgess J O. The wear of polished and glazed zirconia against enamel. *J Prosthet Dent* 2013; **109:** 22-29. 10.1016/S0022-3913(13)60005-0 23328193

[31] Baldissara P, Llukacej A, Ciocca L, Valandro F L, Scotti R. Translucency of zirconia copings made with different CAD/CAM systems. *J Prosthet Dent* 2010; **104:** 6-12. 10.1016/S0022-3913(10)60086-8 20620365

[32] Sawada T, Spintzyk S, Schille C, Schweizer E, Scheideler L, Geis-Gerstorfer J. Influence of Different Framework Designs on the Fracture Properties of Ceria-Stabilized Tetragonal Zirconia/Alumina-Based All-Ceramic Crowns. *Materials (Basel)* 2016; **9:** 339. 10.3390/ma9050339 PMC5503066 28773464

[33] Nakamura K, Harada A, Inagaki R *et al.* Fracture resistance of monolithic zirconia molar crowns with reduced thickness. *Acta Odontol Scand* 2015; **73:** 602-608. 10.3109/00016357.2015.1007479 25635734

[34] Tekin Y H, Hayran Y. Fracture resistance and marginal fit of the zirconia crowns with varied occlusal thickness. *J Adv Prosthodont* 2020; **12:** 283-290. 10.4047/jap.2020.12.5.283 PMC7604235 33149849

[35] Gunge H, Ogino Y, Kihara M, Tsukiyama Y, Koyano K. Retrospective clinical evaluation of posterior monolithic zirconia restorations after 1 to 3.5 years of clinical service. *J Oral Sci* 2018; **60:** 154-158. 10.2334/josnusd.17-0176 29311501

[36] Solá-Ruiz M F, Baixauli-López M, Roig-Vanaclocha A, Amengual-Lorenzo J, Agustín-Panadero R. Prospective study of monolithic zirconia crowns: clinical behavior and survival rate at a 5-year follow-up. *J Prosthodont Res* 2021; **65:** 284-290. 10.2186/jpr.JPR_D_20_00034 33041280

[37] Bömicke W, Rammelsberg P, Stober T, Schmitter M. Short-Term Prospective Clinical Evaluation of Monolithic and Partially Veneered Zirconia Single Crowns. *J Esthet Restor Dent* 2017; **29:** 22-30. 10.1111/jerd.12270 27679981

[38] Weigl P, Sander A, Wu Y, Felber R, Lauer H C, Rosentritt M. In-vitro performance and fracture strength of thin monolithic zirconia crowns. *J Adv Prosthodont* 2018; **10:** 79-84. 10.4047/jap.2018.10.2.79 PMC5917110 29713427

[39] Sorrentino R, Triulzio C, Tricarico M G, Bonadeo G, Gherlone E F, Ferrari M. In vitro analysis of the fracture resistance of CAD-CAM monolithic zirconia molar crowns with different occlusal thickness. *J Mech Behav Biomed Mater* 2016; **61:** 328-333. 10.1016/j.jmbbm.2016.04.014 27104931

[40] Coli P, Karlsson S. Fit of a new pressure-sintered zirconium dioxide coping. *Int J Prosthodont* 2004; **17:** 59-64. 15008234

[41] Abhishek G, Vishwanath S K, Nair A, Prakash N, Chakrabarty A, Malalur A K. Comparative evaluation of bond strength of resin cements with and without 10-methacryloyloxydecyl dihydrogen phosphate (mdp) to zirconia and effect of thermocycling on bond strength - An in vitro study. *J Clin Exp Dent* 2022; **14:** 316-320. 10.4317/jced.59324 PMC9000383 35419176

[42] Scurria M S, Bader J D, Shugars D A. Meta-analysis of fixed partial denture survival: prostheses and abutments. *J Prosthet Dent* 1998; **79:** 459-464. 10.1016/s0022-3913(98)70162-3 9576323

[43] Oh W-S, Anusavice K J. Effect of connector design on the fracture resistance of all-ceramic fixed partial dentures. *J Prosthet Dent* 2002; **87:** 536-542. 10.1067/mpr.2002.123850 12070517

[44] Manicone P F, Rossi Iommetti P, Raffaelli L. An overview of zirconia ceramics: basic properties and clinical applications. *J Dent* 2007; **35:** 819-826. 10.1016/j.jdent.2007.07.008 17825465

[45] Alhusainy A. Zirconia As Dental Restoration Material. *J Pharm Negative Results* 2022; DOI: 10.47750/pnr.2022.13.S09.904.

[46] Gyan Kumar C, Shruthi D P, Sounder Raj K, Kalpana D, Harish G. Zirconia: Substitute for Metal Ceramics. *J Orofac Res* 2014; **4:** 209-212.

[47] Mohsen C A. Evaluation of push-out bond strength of surface treatments of two esthetic posts. *Indian J Dent Res* 2012; **23:** 596-602. 10.4103/0970-9290.107345 23422603

[48] Le Bell-Rönnlöf A M, Lahdenperä M, Lassila L V J, Vallittu P K. Bond strength of composite resin luting cements to fiber-reinforced composite root canal posts. *J Contemp Dent Pract* 2007; **8:** 17-24. 17846667

[49] Jang G W, Kim H S, Choe H C, Son M K. Fracture Strength, Mechanism of Dental Ceramic Crown with Zirconia Thickness. *Procedia Eng* 2011; **10:** 1556-1560.

[50] Kern M, Wegner S M. Bonding to zirconia ceramic: adhesion methods and their durability. *Dent Mater* 1998; **14:** 64-71. 10.1016/s0109-5641(98)00011-6 9972153

[51] Atsu S S, Kilicarslan M A, Kucukesmen H C, Aka P S. Effect of zirconium-oxide ceramic surface treatments on the bond strength to adhesive resin. *J Prosthet Dent* 2006; **95:** 430-436. 10.1016/j.prosdent.2006.03.016 16765155

[52] Warreth A, Elkareimi Y. All-ceramic restorations: A review of the literature. *Saudi Dent J* 2020; **32:** 365-372. 10.1016/j.sdentj.2020.05.004 PMC8461086 34588757

[53] Papaspyridakos P, Bordin T B, Natto Z S. Complications and survival rates of 55 metal-ceramic implant-supported fixed complete-arch prostheses: A cohort study with mean 5-year follow-up. *J Prosthet Dent* 2019; **122:** 441-449. 10.1016/j.prosdent.2019.01.022 30982622

